# Comparative assessment of three drug eluting stents with different platforms but with the same biodegradable polymer and the drug based on quantitative coronary angiography and optical coherence tomography at 12-month follow-up

**DOI:** 10.1007/s10554-017-1251-7

**Published:** 2017-09-30

**Authors:** Robert J. Gil, Jacek Bil, Jacek Legutko, Tomasz Pawłowski, Katarzyna E. Gil, Dariusz Dudek, Ricardo A. Costa

**Affiliations:** 1 Department of Invasive Cardiology, Central Clinical Hospital of the Ministry of Interior and Administration, 137 Woloska Street, 02-507 Warsaw, Poland; 2 Mossakowski Medical Research Centre, Polish Academy of Science, Warsaw, Poland; 3 Institute of Cardiology, Jagiellonian University Medical College, Kraków, Poland; 4 Instituto Dante Pazzanese de Cardiologia, São Paulo, Brazil

**Keywords:** Stent strut thickness, Neointima proliferation, OCT, QCA, Stent strut cross-sectional area

## Abstract

The aim of this study was to compare neointima proliferation in three drug-eluting stents (DES) produced by the same company (Balton, Poland) which are covered with a biodegradable polymer and elute sirolimus (concentration: 1.0 and 1.2 µg/mm^2^), but have different stent platforms and strut thickness: stainless steel Prolim^®^ (115 µm) and BiOSS LIM^®^ (120 µm) and cobalt-chromium Alex^®^ (70 µm). We analyzed data of patients with quantitative coronary angiography (QCA) and optical coherence tomography (OCT) at 12 months from BiOSS LIM Registry, Prolim Registry and Alex OCT clinical trial. There were 56 patients enrolled, in whom 29 Prolim^®^ stents were deployed, in 11—BiOSS LIM^®^ and in 16—Alex stents. The late lumen loss was the smallest in Prolim^®^ subgroup (0.26 ± 0.17 mm) and did not differ from Alex^®^ subgroup (0.28 ± 0.47 mm). This parameter was significantly bigger in BiOSS^®^ subgroup (0.38 ± 0.19 mm; p < 0.05). In OCT analysis there was no statistically significant difference between Prolim^®^ and Alex^®^ subgroups in terms of mean neointima burden (24.6 ± 8.6 vs. 19.27 ± 8.11%) and neointima volume (28.16 ± 15.10 vs. 24.51 ± 17.64 mm^3^). In BiOSS^®^ group mean neointima burden (30.9 ± 6.2%) and mean neointima volume (44.9 ± 4.9 mm^3^) were significantly larger. The morphological analysis revealed that in most cases in all groups the neointima was homogenous with plaque presence only around stent struts. In the QCA and OCT analysis regular DES (Prolim^®^ and Alex^®^) obtained similar results, whereas more pronounced response from the vessel wall was found in the BiOSS^®^ subgroup.

## Introduction

Drug eluting stents (DES) reduce the incidence of restenosis and thereby also the incidence of repeated revascularizations. Initially, most stents were made of stainless steel and therefore had relatively thick struts. Today the world applies the platinum-chromium or cobalt-chromium alloys as the stent platform, what makes possible to produce much thinner struts (even half smaller). The ISAR-STEREO trial demonstrated that a thin-strut stent (≤ 100 µm) had a lower rate of restenosis than a thick strut stent (> 100 µm) of similar design [1]. Comparable results were found in several other trials [2–4]. The abovementioned studies suggested that thick struts might predispose to excessive neointima proliferation, however, these results are based on studies with bare metal stents or the first generation DES presently not available on the market.

The deployment of a durable polymer DES (DP-DES) is a standard of care in patients with coronary artery lesions. However, studies assessing biodegradable polymer DES (BP-DES) proved the non-inferiority to DP-DES with the expectation for the decreased inflammatory response after stent implantation and, in consequence, for faster vessel healing [5].

The aim of this study was to compare neointima proliferation in three DES produced by the same company (Balton, Poland) which are covered with the same biodegradable polymer and elute the same drug (sirolimus concentrations: 1.0 µg/mm^2^ for Alex^®^ and BiOSS LIM^®^, while 1.2 µg/mm^2^ Prolim^®^) but have different stent platforms: stainless steel (Prolim^®^, BiOSS LIM^®^) or cobalt-chromium (Alex^®^) and strut thickness: 115, 120 and 70 µm, respectively.

## Materials and methods

### Study population and study design

We included patients who had implanted one of the following stents (Prolim^®^, Alex^®^ or BiOSS LIM^®^) and had performed optical coherence tomography (OCT) during 12-month angiographic follow-up. Patients participated in one of the following studies: Prolim Registry, BiOSS LIM Registry or Alex OCT study. The detailed inclusion and exclusion criteria are described elsewhere [6–8]. The appropriate Ethics Committees approved study protocols.

### Study device

The BiOSS LIM^®^ is a coronary, dedicated bifurcation balloon-expandable stent made of 316L stainless steel (strut thickness 120 μm; strut width 180 μm). The cover ratio is 18%. The stent consists of two parts, proximal and distal, joined with two connecting struts (depending on stent size: 1.8–2.3 mm in length) at the step-up middle zone. The proximal part of the stent has a larger diameter in relation to the distal part (diameter ratio of proximal to distal parts is included between 1.15 and 1.3). Maximal diameter of expanded stent cell is 3.5 mm [8].

The Prolim^®^ stent is a balloon expandable coronary stent with RX delivery system. The stent platform is made of a laser-cut 316L metallic tube with a wall thickness of 115 µm and strut width of 80 µm. The cover ratio is 19%. Maximal diameter of expanded stent cell is 1.8 mm [9].

The Alex^®^ stent is a balloon expandable coronary stent with RX delivery system. The stent platform is made of a laser-cut cobalt-chromium tube with a wall thickness of 70 µm and strut width of 75 µm. The cover ratio is 18%. Maximal diameter of expanded stent cell is 1.5 mm [9].

All abovementioned stents are covered with a mixture of biodegradable poly(lactide-co-glycolide) copolymer and an antiproliferative substance—sirolimus. The polymer layers release sirolimus (1.0 µg/mm^2^ for Alex and BiOSS LIM and 1.2 µg/mm^2^ for Prolim) in a time-controlled process due to their biodegradation (lasting around 8 weeks) [10].

### Procedure

Percutaneous coronary interventions (PCI) were performed according to local standards via radial or femoral access using 6 Fr or 7 Fr guiding catheters. Pharmacological treatment was according to the most recent guidelines [11]. Troponin I (TnI), creatine kinase (CK) and creatine kinase-myocardial band (CK-MB) were measured pre-procedural, 6 and 24 h after procedure in all patients. Periprocedural myocardial infarction (type 4a) was defined according to the third universal definition [12].

### Follow-up

The assessment of the anginal status, data collection of adverse events, details of any subsequent coronary interventions, and the use and changes in concomitant medications were collected at 12 ± 0.5 months. The angiographic control was planned at 12 months, in which patients in Prolim and BiOSS LIM Registries had OCT examination randomly (approximately 15% of patients), whereas in the Alex OCT study it was mandatory at 12 months.

### Endpoints

The primary endpoint was the cumulative rate of major adverse cardiovascular events (MACE) consisting of cardiac death, myocardial infarction (MI) and clinically-driven target lesion revascularization (TLR). Secondary endpoints included cardiac death, all-cause death, MI, TLR, stent thrombosis, late lumen loss (LLL) assessed in quantitative coronary angiography (QCA), the percentage of covered struts and neointima volume and morphology characteristics assessed in OCT as well as the device success rate. Cardiac death included death resulting from an acute MI, sudden cardiac death, death due to heart failure and death due to cardiac procedures. All deaths were deemed cardiac unless proven otherwise. MI was defined according to third universal definition [12]. Clinically-driven TLR was defined as reintervention of the target lesion due to presence of a symptomatic ≥ 50% diameter stenosis during follow-up. Device success was defined as successful deployment of the intended stent in the target site without a system failure. The definite stent thrombosis was defined as state with symptoms suggestive of an acute coronary syndrome and angiographic or pathologic confirmation of stent thrombosis. The probable stent thrombosis was defined as the unexplained death within 30 days or target vessel myocardial infarction without angiographic confirmation of stent thrombosis, and the possible stent thrombosis was defined as any unexplained death after 30 days [13].

### Quantitative angiography analysis

All coronary angiograms were recorded after intracoronary administration of 200 μg of nitroglycerin. Two orthogonal views were chosen to visualize the target lesion. A QCA analysis was performed using commercially available software (QCA-CMS version 5.0, Medis, Leiden, the Netherlands). Catheter calibration was used in all cases. The following parameters: lesion length, reference vessel diameter, minimal lumen diameter, % diameter stenosis, acute lumen gain and LLL were calculated as described previously [14].

### Optical coherent tomography analysis

Briefly, after wiring the artery with the guidewire as described previously, the Dragon Fly catheter (LigthLab Co.) was advanced distally to the implanted stent and during continuous contrast media flush (Iodixanol, Visipaque GE Healthcare), the automatic pullback was performed. The commercially available console (M2 or M3 by LigthLab Co.) was used. Optical coherence tomography images were obtained along the region of interest, which was the implanted stent plus 5 mm both proximal and distal. Off-line analysis was performed after careful recalibration of acquired images along the reconstructed longitudinal segment. Calibration was obtained by adjusting the z-offset, the zero-point setting of the system. The analysis was performed applying a dedicated off-line software (St Jude Medical). Quantitative measurements of the minimal lumen area and minimal lumen diameter were obtained in all consecutive frames along the region of interest using semi-automated algorithm. Additionally, the mean value of all lumen area cross-sections measured inside the region was calculated. Additionally, lumen volume analysis was performed along region of interest—all measured lumen area cross-sections were summed. Mean neointimal burden was calculated as the ratio of the mean neointima area to the mean stent area [7, 15].

Moreover, to assess stent apposition OCT analysis was performed every 0.2 mm of the stent. The stent struts apposition was classified as: (1) apposed (2) protruded and (3) malapposed according to a distance length between vessel wall and center of the stent strut. If such distance was: (1) more than 130 μm, malapposition was detected, (2) in range of 20 to 130 μm, protrusion was detected. The morphology of the neointima was analyzed according to the previously validated OCT criteria, and classified as type I (thin cap neoatheroma, lipid-rich), type II (thick-cap, layered), type III (peri-strut, homogenous) and type IV (pre-existing, homogenous) [16, 17].

### Statistical analysis

Continuous variables were presented as mean ± standard deviation. Categorical data were presented as numbers (%). Continuous variables were compared using an ANOVA test, and categorical data using the χ^2^ test. If distribution was not normal (verified with the Shapiro–Wilk test) for continuous variables Kruskal–Wallis test was used. P values of < 0.05 were considered statistically significant. If P was < 0.05 for determining the statistical significance between groups appropriate post-hoc tests were used. Pearson correlation was applied in continuous variables. Additionally, univariate and multivariate linear regression analyses were performed. Statistical analyses were performed using R 3.0.2 for OS (R Foundation, Vienna, Austria).

## Results

### Baseline clinical and angiographic characteristics

A total of 56 patients were enrolled into this analysis, i.e. 11 patients—with BiOSS LIM^®^ stent implanted, 29 patients with Prolim^®^, and 16 patients—with Alex^®^ stent deployed. The baseline characteristics was comparable between groups, apart from the age. The mean age was significantly higher in the Prolim^®^ subgroup than in the BiOSS^®^ subgroup (68 ± 10 vs. 60 ± 6 years, p < 0.05). The mean age in Alex group was 62 ± 9 years. The detailed clinical characteristics is presented in Table 1.

**Table 1:** Baseline clinical characteristics

Baseline clinical characteristics	BiOSS LIM^®^	Prolim^®^	Alex^®^	P
	n = 11 (%)	n = 29 (%)	n = 16 (%)	
Age (years)	60 ± 6	68 ± 10	62 ± 9	0.03*
Women	3 (27)	9 (31.0)	4 (25)	0.96
Hypertension	8 (72.7)	25 (86.2)	12 (75)	0.63
Hypercholesterolemia	11 (100)	24 (82.8)	9 (56.3)	0.13
Diabetes type 2	3 (27.3)	9 (31.0)	7 (43.8)	0.65
Prior MI	6 (54.5)	7 (24.1)	9 (56.3)	0.11
Prior PCI	3 (27.3)	8 (27.6)	6 (37.5)	0.79
CABG	0	2 (6.7)	0	0.9
Chronic kidney disease	0	3 (10.3)	0	0.8
Clinical indication for PCI				
Planned PCI	11 (100)	19 (65.5)	10 (62.5)	0.10
UA	0	6 (20.7)	6 (37.5)	0.24
NSTEMI	0	4 (13.8)	0	0.67
STEMI	0	0	0	0.99

*MI* myocardial infarction, *PCI* percutaneous coronary intervention, *CABG* coronary artery bypass graft, *UA* unstable angina, *NSTEMI* non-ST-elevation myocardial infarction, *STEMI* ST-elevation myocardial infarction; *p < 0.05 for Prolim vs. BiOSS LIM

In the Prolim^®^ and BiOSS LIM^®^ subgroups most patients presented with multivessel disease and in all groups lesions were of the moderate complexity. In the Prolim^®^ and BiOSS^®^ subgroups lesions were located most frequently in left anterior descending artery, 48.3 and 72.7%, respectively. In the Alex^®^ subgroup the left circumflex artery was the most frequently stented vessel (43.8%). More details are presented in Table 2.

**Table 2:** Baseline angiographic characteristics

Baseline angiographic characteristics	BIOSS LIM^®^	Prolim^®^	Alex^®^	P
	n = 11 (%)	n = 29 (%)	n = 16 (%)	
Multivessel disease	7 (63.6)	18 (62.1)	0	0.0014**^,^***
Lesion type				
A	0	9 (31.0)	0	0.13
B1 (27.3)	3 (27.3)	17 (58.6)	5 (31.3)	0.15
B2 (54.5)	6 (54.5)	2 (6.9)	7 (43.8)	0.027*^,^**
C	2 (18.2)	1 (3.5)	4 (25)	0.45
Lesion location				
LM	0	0	0	0.98
LAD	8 (72.7)	14 (48.3)	6 (37.5)	0.31
LCx	3 (27.3)	5 (17.2)	7 (43.8)	0.31
RCA	0	10 (34.5)	3 (18.8)	0.23
Bifurcation lesions				
Side branch > 2 mm	10 (90.9)	4 (13.8)	7 (43.8)	0.0006*^,^**^,^***
Side branch < 2 mm	1 (9.1)	5 (17.2)	NA	0.25
None	0	20 (68.9)	9 (56.2)	0.005*^,^***
Vessel tortuosity				
None—mild	7 (63.6)	17 (58.6)	9 (56.2)	0.84
Moderate—severe	4 (36.4)	12 (41.4)	7 (43.8)	0.91
Calcification				
None—mild	8 (72.7)	20 (68.9)	13 (81.2)	0.87
Moderate—severe	3 (27.3)	9 (31.1)	3 (18.8)	0.85

*LAD* left anterior descending artery, *LM* left main stem, *LCx* left circumflex artery, *RCA* right coronary artery, *p < 0.05 for Prolim vs. BiOSS LIM; **p < 0.05 Prolim vs. Alex; ***p < 0.05 BiOSS LIM vs. Alex

### Procedural characteristics

The main procedural variables are presented in Table 3. The device success rate was 100% in all subgroups. There were no significant differences in procedural details as well as in the rate of periprocedural complications in those three groups.

**Table 3:** Procedural characteristics

Procedural characteristics	BIOSS LIM^®^	Prolim^®^	Alex^®^	P
	n = 11 (%)	n = 29 (%)	n = 16 (%)	
Device success	11 (100)	29 (100)	16 (100)	0.98
Predilatation	5 (45.5)	17 (58.6)	4 (25)	0.16
Postdilatation	2 (18.2)	8 (27.6)	4 (25)	0.90
Nominal stent diameter (mm)	3.57 ± 0.12 × 3.0 ± 0.05	3.25 ± 0.42	3.25 ± 0.35	0.7
Nominal stent length (mm)	17.45 ± 1.21	13.67 ± 2.88	16.41 ± 5.95	0.04*
Stent maximal inflation pressure (atm)	13.34 ± 1.98	15.33 ± 2.24	14.5 ± 1.8	0.56
Balloon to artery ratio	1.11 ± 0.05	1.09 ± 0.08	1.08 ± 0.09	0.87
Additional stent implantation due to dissection	0	0	0	0.98
Additional stent implantation due to lesion length	0	0	0	0.98

*p < 0.05 for Prolim vs. BiOSS LIM

### Clinical outcomes

The clinical follow-up at 12 months was available in all patients. The MACE rate was: 9.1, 0 and 6.25% in the BiOSS LIM^®^, Prolim^®^ and Alex^®^ subgroups, respectively. In the observation there was one case of TLR treated with another DES in the BiOSS^®^ subgroup as well as in the Alex^®^ subgroup. There was no death or stent thrombosis.

### Quantitative coronary angiography and optical coherence tomography analysis

**Fig. 1:**
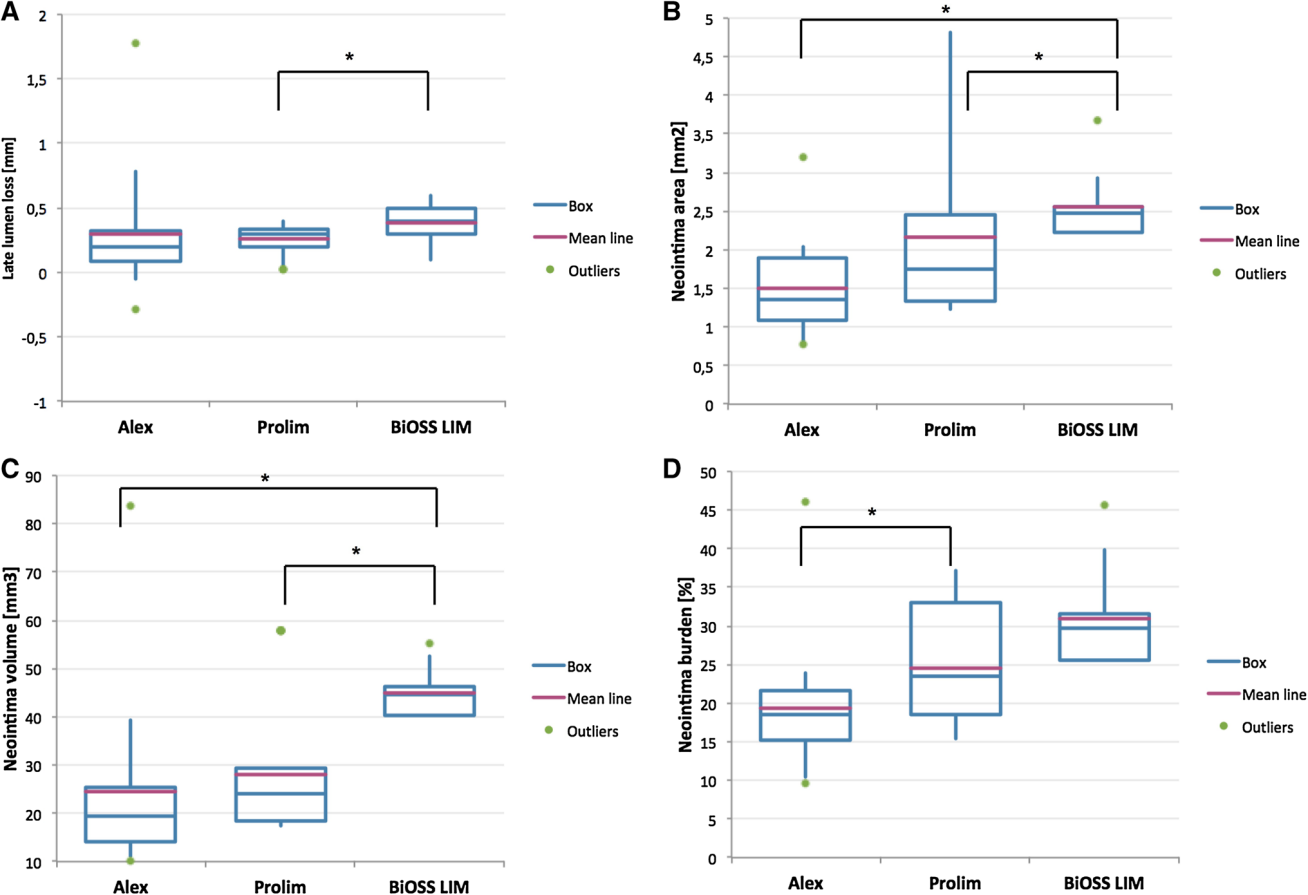
QCA and OCT assessment **a** late lumen loss, **b** neointima area, **c** neointima volume, **d** neointima burden

The QCA data are presented in Table 4. The immediate angiographic success rate was 100%. Acute lumen gain was the lowest in the BiOSS^®^ subgroup (1.35 ± 0.23 mm) and significantly differed from acute lumen gain in the Prolim^®^ group (1.86 ± 0.39 mm) as well as in the Alex^®^ subgroup (1.78 ± 0.47 mm). Whereas the late lumen loss was the smallest in the Prolim^®^ subgroup (0.26 ± 0.17 mm) and it was significantly lower than in the BiOSS^®^ subgroup (0.38 ± 0.19 mm). The LLL in the Alex^®^ subgroup was 0.28 ± 0.47 mm, but due to relatively high standard deviation (among others due to one case of TLR) it did not differ significantly between the other two subgroups (Fig. 1a). Worth mentioning is the fact that when analyzing the BiOSS LIM^®^ stent as two parts with different diameter (3.57 ± 0.12 × 3.0 ± 0.05 mm) we obtained the LLL in the proximal part of 0.36 ± 0.25 mm, and in the distal part of 0.39 ± 0.13 mm (p = NS).

**Table 4:** Quantitative coronary angiographic analysis

Parameter	BiOSS LIM^®^			Prolim^®^	Alex^®^	
	n = 11 (%)			n = 29 (%)	n = 16 (%)	
	MV	MB	Mean			
Pre stenting						
Lesion length (mm)	15.86 ± 2.65			14.47 ± 1.84	11.75 ± 3.29	0.001**^,^***
RVD (mm)	3.69 ± 0.16	3.0 ± 0.13	3.35 ± 0.14	3.39 ± 0.24	2.93 ± 0.34	p < 0.01**^,^***
MV—%DS	53.1 ± 9.7	44.8 ± 12.1	48.9 ± 10.9	64.6 ± 15.3	61.6 ± 11.4	0.0002**^,^****
MLD (mm)	1.73 ± 0.27	1.65 ± 0.14	1.69 ± 0.2	1.21 ± 0.34	1.11 ± 0.32	0.00008*^,^***
Post stenting						
RVD (mm)	3.68 ± 0.19	3.02 ± 0.08	3.35 ± 0.14	3.41 ± 0.23	3.06 ± 0.4	p < 0.01**^,^***
MV—%DS	9.4 ± 4.3	8.8 ± 3.4	9.1 ± 7.7	9.6 ± 4.1	5.31 ± 4.33	0.02**
MLD (mm)	3.33 ± 0.17	2.75 ± 0.1	3.04 ± 0.14	3.08 ± 0.28	2.89 ± 0.4	0.03**
ALG (mm)	1.6 ± 0.3	1.1 ± 0.16	1.35 ± 0.23	1.86 ± 0.39	1.78 ± 0.47	0.001*^,^***
Follow-up						
RVD (mm)	3.63 ± 0.25	2.96 ± 0.09	3.29 ± 0.17	3.45 ± 0.25	2.98 ± 0.45	p < 0.01**^,^***
MV—%DS	18.2 ± 4.3	20.3 ± 7.4	19.3 ± 11.7	18.3 ± 6	12.69 ± 17.99	p = 0.08
MLD (mm)	2.97 ± 0.25	2.36 ± 0.22	2.67 ± 0.23	2.82 ± 0.37	2.61 ± 0.69	p = 0.03**
LLL (mm)	0.36 ± 0.25	0.39 ± 0.13	0.38 ± 0.19	0.26 ± 0.17	0.28 ± 0.47	p = 0.02*

*RVD* reference vessel diameter, *%DS* % diameter stenosis, *MLD* minimal lumen diameter, *ALG* acute lumen gain, *LLL* late lumen loss, *FU* follow-up, *MV* main vessel, *MB* main branch, *p < 0.05 for Prolim vs. BiOSS LIM; **p < 0.05 Prolim vs. Alex; ***p < 0.05 BiOSS LIM vs. Alex

**Fig. 2:**
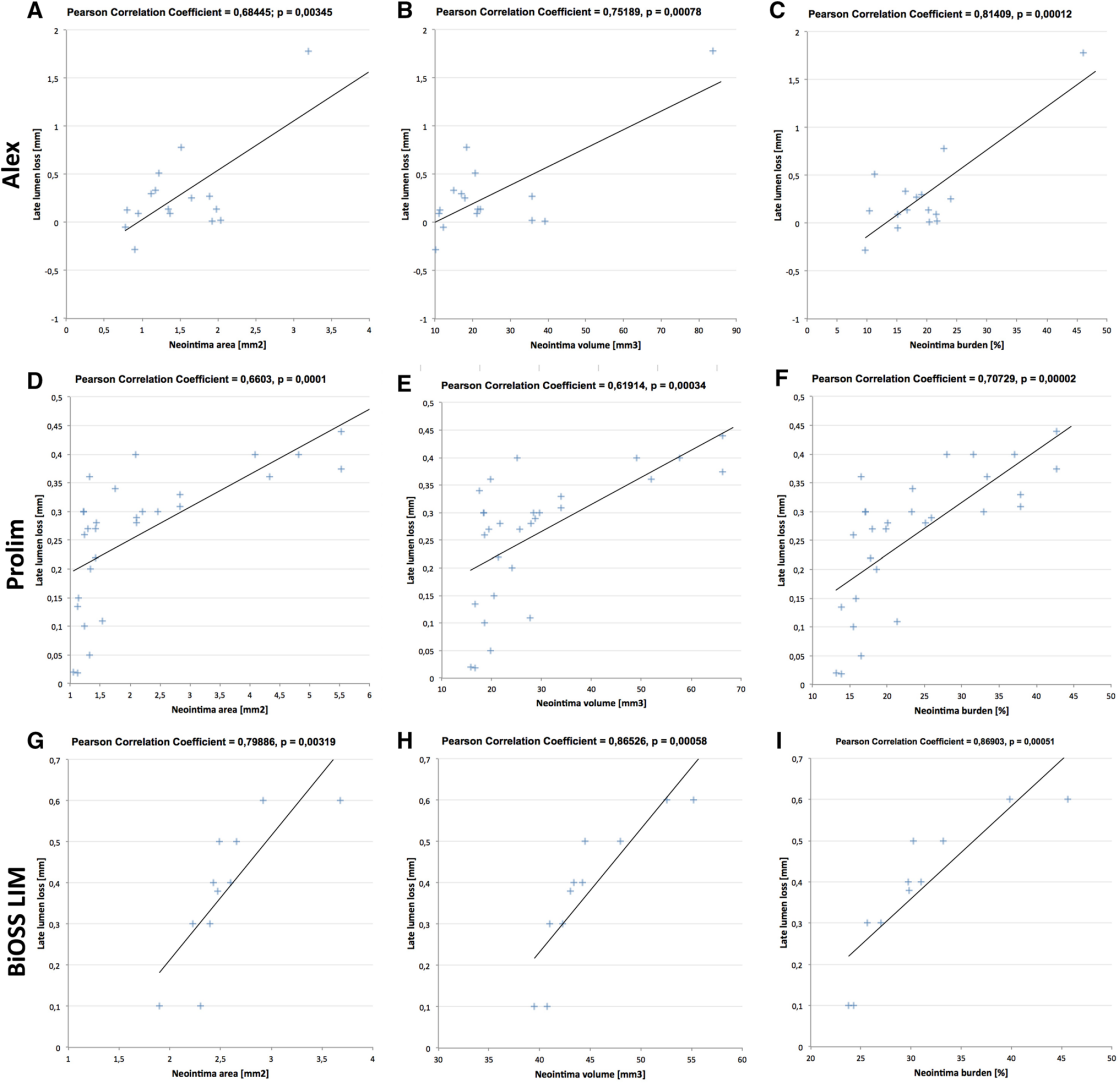
Correlation analysis between QCA and OCT parameters for Alex, Prolim and BiOSS LIM stents

The OCT analysis data are presented in Table 5. The OCT at 12 months was performed in all patients. The rate of embedded stent struts was comparable between Prolim^®^ and Alex^®^ subgroups (98.6 and 99.2%), and was significantly lower in the BiOSS^®^ subgroup (95.8%). Similarly, there was no statistically significant difference between Prolim^®^ and Alex^®^ subgroups in terms of mean neointima burden (24.6 ± 8.6 vs. 19.27 ± 8.11%) and neointima volume (28.16 ± 15.10 vs. 24.51 ± 17.64 mm^3^), while in BiOSS^®^ group these parameters were significantly larger, 30.9 ± 6.2% and 44.9 ± 4.9 mm^3^ (Fig. 1b–d). Moreover, as shown on the Fig. 2 in each group LLL values significantly correlated with OCT parameters: neointima area, neointima volume and neointima burden.

**Table 5:** Optical coherence tomography analysis at 12 months

Stent type	BiOSS LIM^®^	Prolim^®^	Alex^®^	
Parameter	n = 11 (%)	n = 29 (%)	n = 16 (%)	
Stent apposition				
Embedded	95.8	98.6	99.2	0.00001*^,^***
Protruding	2.5	1.2	0.8	0.0054*^,^***
Uncovered	1.3	0.07	0.02	0.002*^,^***
Malapposed	0.4	0.1	0	0.45
OCT parameters				
Mean minimal lumen area (mm^2^)	3.72 ± 0.57	4.82 ± 1.41	5.22 ± 1.95	0.027***
Mean lumen area (mm^2^)	5.76 ± 0.73	6.21 ± 1.10	6.46 ± 1.8	0.35
Mean stent area (mm^2^)	8.31 ± 0.4	8.39 ± 2.26	7.94 ± 1.84	0.7
Mean neointima area (mm^2^)	2.55 ± 0.41	2.17 ± 0.37	1.49 ± 0.60	0.012*^,^***
Neointima volume (mm^3^)	44.9 ± 4.9	28.16 ± 15.10	24.51 ± 17.64	0.0008*^,^***
Mean neointima burden (%)	30.9 ± 6.2	24.6 ± 8.6	19.27 ± 8.11	0.0009***
Neoatherosclerosis assessment				
Type I (thin cap, lipid-rich)	1 (9.1)	1 (3.4)	0	0.92
Type II (thick cap, layered)	2 (18.2)	4 (13.8)	2 (12.5)	0.97
Type III (peristrut, homogenous)	6 (54.5)	10 (34.5)	3 (18.8)	0.25
Type IV (preexisting, homogenous)	2 (18.2)	14 (48.3)	11 (68.7)	0.09

*p < 0.05 for Prolim vs. BiOSS LIM; **p < 0.05 Prolim vs. Alex; ***p < 0.05 BiOSS LIM vs. Alex

The morphological analysis revealed that in most cases in all groups the neointima was homogenous with plaque presence only around the stent struts. Patterns did not differ significantly (Table 5).

In Tables 6 and 7 there are presented linear regression analyses for LLL value and for neointima burden value, respectively. Regarding LLL stent length was the only significantly correlating value both in univariate (regression coefficient − 0.021, 95% CI − 0.040 to − 0.002, p = 0.03) as well as in multivariate analysis (− 0.025, 95% CI − 0.046 to − 0.004, p = 0.02). Whereas when neointima burden was analyzed the following factors were significant in the univariate analysis: strut width (87.676, 95% CI 30.305–145.048, p = 0.003) and strut thickness (151.185, 95% CI 38.862–263.508, p = 0.009) as well as in the multivariate analysis: strut width (66.406, 95% CI − 1.957 to 134.768, p = 0.04) and postdilatation (− 4.860, 95% CI − 9.982 to 0.263, p = 0.04).

**Table 6:** Late lumen loss—linear regression

Variate	Linear regression coefficient, 95% CI, p	
	Univariate analysis	Multivariate analysis
Strut width	1.082 (− 0.706 to 2.870), p = 0.23	1.632 (− 0.488 to 3.753), p = 0.13
Strut thickness	0.443 (− 3.044 to 3.930), p = 0.80	− 1.568 (− 5.935 to 2.799), p = 0.47
Strut cross-sectional area	1.381 (0.403–5.212), p = 0.16	2.111 (1.444–2.993), p = 0.03
Stent diameter	− 0.174 (− 0.432 to 0.084), p = 0.18	− 0.225 (− 0.490 to 0.039), p = 0.09
Stent length	− 0.021 (− 0.040 to − 0.002), p = 0.03	− 0.025 (− 0.046 to − 0.004), p = 0.02
Predilatation	0.092 (− 0.052 to 0.237), p = 0.21	0.111 (− 0.044 to 0.265), p = 0.16
Postdilatation	− 0.000 (− 0.160 to 0.159), p = 0.97	− 0.067 (− 0.226 to 0.092), p = 0.40
Diabetes	0.003 (− 0.152 to 0.158), p = 0.97	0.037 (− 0.136 to 0.209), p = 0.67
Arterial hypertension	− 0.073 (− 0.256 to 0.111), p = 0.43	− 0.100 (− 0.354 to 0.153), p = 0.43
Dyslipidemia	− 0.019 (− 0.198 to 0.159), p = 0.83	0.025 (− 0.254 to 0.305), p = 0.86

**Table 7:** Neointima burden—linear regression

Variate	Linear regression coefficient, 95% CI, p	
	Univariate analysis	Multivariate analysis
Strut width	87.676 (30.305 to 145.048), p = 0.003	66.406 (− 1.957 to 134.768), p = 0.04
Strut thickness	151.185 (38.862 to 263.508), p = 0.009	68.154 (− 72.634 to 208.942), p = 0.34
Strut cross-sectional area	90.452 (45.211–134.749), p = 0.001	87.198 (3.334–147.348), p = 0.01
Stent diameter	− 3.712 (− 12.657 to 5.233), p = 0.41	− 7.757 (− 16.276 to 0.762), p = 0.07
Stent length	− 0.348 (− 1.030 to 0.334), p = 0.31	− 0.466 (− 1.135 to 0.202), p = 0.17
Predilatation	2.750 (− 2.240 to 7.741), p = 0.27	1.725 (− 3.254 to 6.704), p = 0.49
Postdilatation	− 2.069 (− 7.513 to 3.376), p = 0.45	− 4.860 (− 9.982 to 0.263), p = 0.04
Diabetes	− 0.669 (− 5.982 to 4.644), p = 0.80	− 0.416 (− 5.985 to 5.153), p = 0.88
Arterial hypertension	− 0.553 (− 6.887 to 5.780), p = 0.86	− 4.343 (− 12.510 to 3.825), p = 0.29
Dyslipidemia	4.950 (− 1.033 to 10.934), p = 0.10	5.426 (− 3.581 to 14.432), p = 0.23

### The new cut-off of real thin-strut stents

**Fig. 3:**
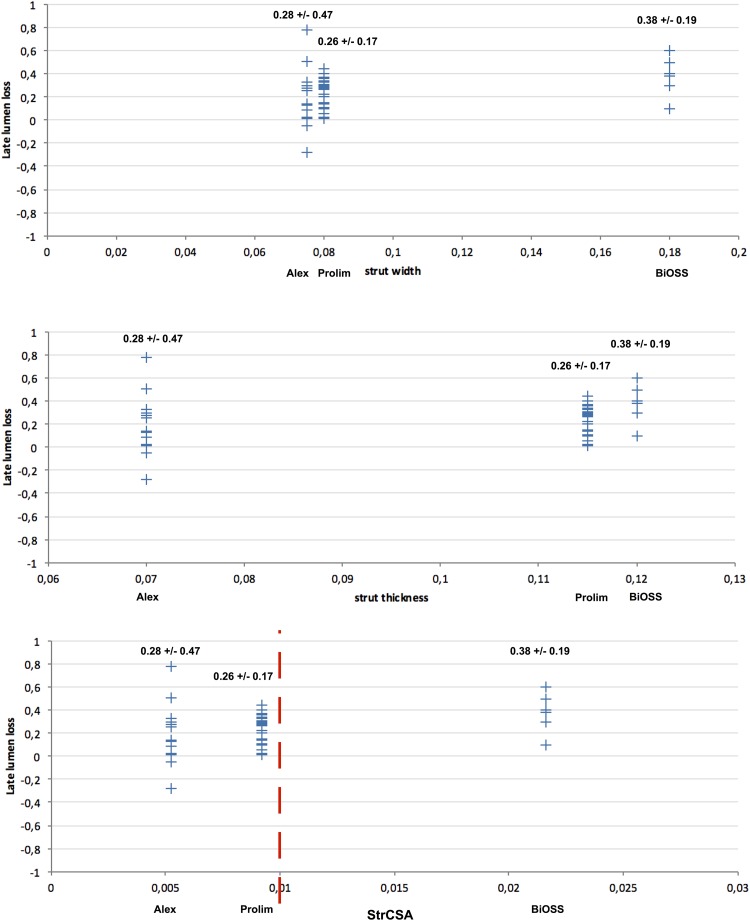
Correlation analysis between late lumen loss and stent parameters (strut width, strut thickness, and strut cross-sectional area) for Alex, Prolim and BiOSS LIM stents

**Fig. 4:**
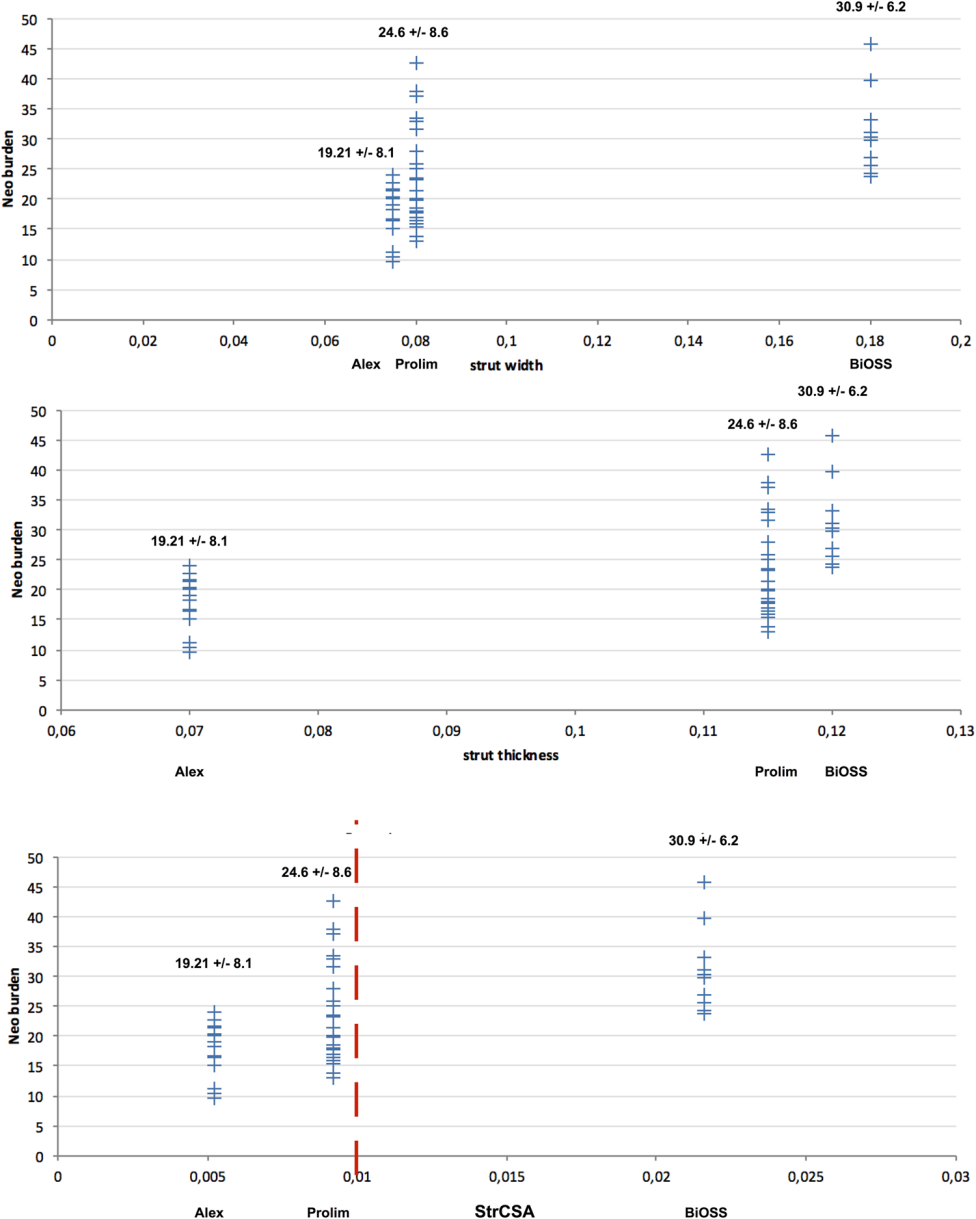
Correlation analysis between neointima burden and stent parameters (strut width, strut thickness, and strut cross-sectional area) for Alex, Prolim and BiOSS LIM stents

When analyzing the abovementioned parameters we decided to verify the hypothesis that not only strut thickness as one strut dimension is responsible for vascular response, but 2-dimensional parameter is more accurate (Figs. 3, 4). Therefore, we have introduced a new parameter (strut cross-sectional area—StrCSA) that is the product of strut width and its thickness. The following values were obtained: for Alex^®^—0.005250 mm^2^, for Prolim^®^—0.009200 mm^2^, and for BiOSS LIM^®^—0.021600 mm^2^.

## Discussion

The LLL is a parameter widely used for assessment of the stent’s performance. We found that it was comparable in the Alex^®^ and Prolim^®^ subgroups (0.28 ± 0.47 and 0.26 ± 0.17 mm, respectively), whereas it was significantly bigger in the BiOSS^®^ subgroup (0.38 ± 0.19 mm, p < 0.05). It is worth stressing that there was no difference between LLL values for proximal and distal part of BiOSS^®^ LIM stent. The lack of differences between Alex^®^ and Prolim^®^ may be at least partly explained by higher sirolimus concentration on this second stent (1.2 vs. 1.0 µg/mm^2^). If we take into account the same biodegradable polymer one can say that sirolimus in higher concentration reduced the effect of thicker struts of Prolim^®^ stent.

The obtained results were better than those observed in Cypher^®^ stent (0.40 ± 0.65 mm, strut thickness 140 µm) [18], but worse than in the newest generation stents: for Excel II^®^ stent − 0.12 ± 0.34 mm (strut thickness 88 µm), for Orsiro^®^ stent—0.10 ± 0.32 mm (strut thickness 60 µm), for Xience^®^ stent—0.11 ± 0.29 mm (strut thickness 80 µm), and for Supralimus^®^ stent—0.09 ± 0.37 mm (strut thickness 66 µm) [19–21]. It should be emphasized that the highest sirolimus concentration was for Cypher^®^ comparing with Orsiro^®^ and Supralimus^®^ stents (1.4, 1.2 and 1.2 µg/mm^2^, respectively).

In our study regarding LLL stent length was the only significantly correlating value both in univariate (regression coefficient − 0.021, 95% CI − 0.040 to − 0.002, p = 0.03) as well as in multivariate analysis (− 0.025, 95% CI − 0.046 to − 0.004, p = 0.02).

There are many factors influencing on the vessel wall response to stent implantation. There is not only strut thickness and the drug’s type but also the type of stent platform and drug’s carrier, drug itself (including concentration) as well as accompanying diseases (such as diabetes mellitus). Strut width is rather forgotten and generally not analyzed parameter, because in most contemporary stents the cross-sectional area of the strut has the shape of the circle or square (width = thickness). This is a mistake in our opinion. After all, strut width determines the area of stent adhering to the wall that initiates vascular response, while strut thickness is probably responsible for the duration of neointima proliferation. Therefore, it is rationale that for standard vascular response assessment one should use the “product” of these two parameters (e.g. StrCSA) as better illustrating the geometric form of the stent strut and its potential impact on neointimal proliferation magnitude.

**Fig. 5:**
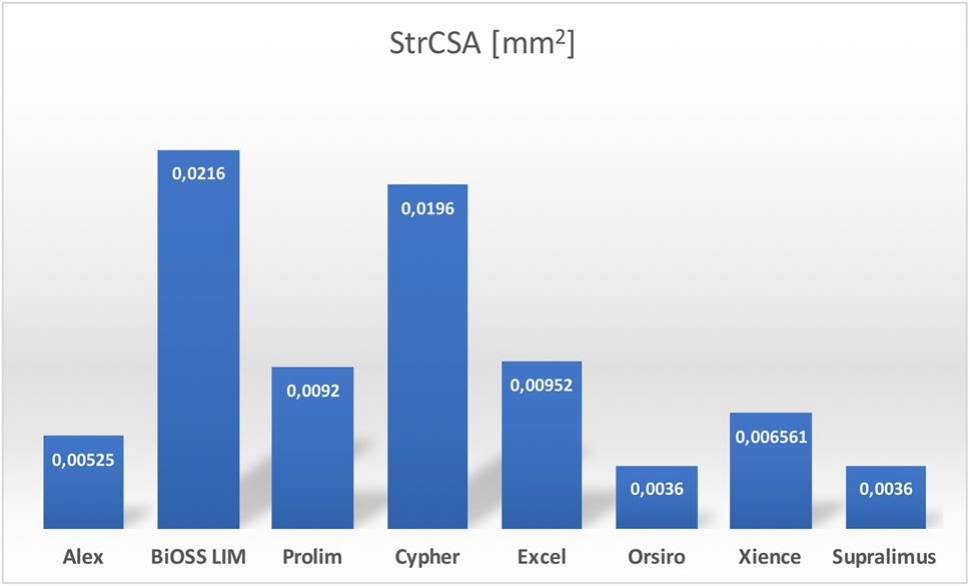
Strut cross-sectional area for commonly available drug-eluting stents

The analysis of the proposed parameter for all analyzed stents entitled to presume that its relatively low LLL value for Prolim^®^ stent was associated with the smaller value of strut width compared with BiOSS LIM^®^ (Alex^®^—75 µm, Prolim^®^—80 µm, BiOSS LIM^®^—180 µm). StrCSA illustrates differences between the analyzed stents in terms of strut’s geometry even better (Alex^®^—0.005250 mm^2^, Prolim^®^—0.009200 mm^2^, BiOSS LIM^®^—0.021600 mm^2^). Interesting conclusions could also be drawn based on the analysis of the StrCSA calculated for other well-known stents on the market. The following values were obtained: Cypher^®^—0.019600 mm^2^, Excel^®^—0.009520 mm^2^, Orsiro^®^—0.003600 mm^2^, Xience^®^—0.006561 mm^2^ and Supralimus^®^—0.003600 mm^2^. This calculation definitely shows that the higher the StrCSA is, the bigger LLL value are obtained (Fig. 5).

OCT enabling single stent strut analysis seems to be the best method for tissue response assessment after stent implantation. We found almost complete vessel healing 12 months after the index procedure for all assessed stents, however stent strut coverage was significantly worse for BiOSS LIM^®^ stent compared with others (Alex^®^ 99.2%, Prolim^®^ 98.6%, BiOSS LIM^®^ 95.8%,). It is very likely that the value of BiOSS LIM^®^ strut coverage rate was determined not only by strut thickness itself but by coronary bifurcation as well. It is worth to be stressed that this parameter for Alex^®^ and Prolim^®^ stents was superior to everolimus-eluting stent Xience V^®^ (Abbott Vascular, Santa Clara, CA) and zotarolimus-eluting stent Resolute Integrity^®^ (96.5 and 93.5%, respectively) in the 13-month OCT substudy of the RESOLUTE All Comers trial [22]. On the other hand, OCT substudy of LEADERS trial showed that BP-DES (BioMatrix, strut thickness—112 µm) characterized a more complete stent coverage (99.4%) as compared with DP-DES—Cypher (97.9%) at 9 months follow-up [23]. Really, there is no clear and simple way to assess vessel wall response after stent implantation even with OCT use. There are no doubts that such a healing process is multifactorial.

As mentioned earlier there was no statistically significant difference between Prolim^®^ and Alex^®^ subgroups in terms of mean neointima burden (24.6 ± 8.6 vs. 19.27 ± 8.11%) and neointima volume (28.16 ± 15.10 vs. 24.51 ± 17.64 mm^3^), while in BiOSS^®^ group these parameters were significantly larger (30.9 ± 6.2% and 44.9 ± 4.9 mm^3^). This is a strong confirmation that the higher sirolimus concentration and small stent width (and StrCSA in consequence) in Prolim^®^ stent are responsible for that. Values obtained for Prolim^®^ and Alex^®^ subgroups were higher comparing with cobalt-chromium Excel II stent with strut thickness of 88 µm (11.93 ± 6.08 mm^3^ and 6.77 ± 4.14%) [24]. Interestingly, these values were comparable to those obtained in Cypher stent (26.61 ± 23.06 mm^3^ and 15 ± 8%) [25]. We believe that higher sirolimus concentration on Cypher stent (1.4 μg/mm^2^) and the related drug potency explains those differences.

In our study when neointima burden was analyzed the following factors were significant in the univariate analysis: strut width (87.676, 95% CI 30.305–145.048, p = 0.003) and strut thickness (151.185, 95% CI 38.862–263.508, p = 0.009) as well as in the multivariate analysis: strut width (66.406, 95% CI − 1.957 to 134.768, p = 0.04) and postdilatation (− 4.860, 95% CI − 9.982 to 0.263, p = 0.04).

Although it is often thought that OCT is the most accurate technique for analyzing coronary lesions, whilst QCA incurs systematic underestimation [26], in our study we have shown that in all three stents there was a strong correlation between LLL and OCT parameters such as neointima area, neointima volume and neointima burden (Fig. 2). Also, the role of StrCSA were confirmed in OCT imaging. The higher the “product” was, the bigger neointima burden and neointima volume were obtained (Fig. 4; Tables 6, 7).

We strongly believe that the proposed new parameter might be clinically relevant, however needs to be evaluated in a properly designed prospective study.

Ultimately, it is worth mentioning the OCT results which provided the additional insight in the characteristics of neointima formation. In most cases homogenous peristrut or preexisting atheroma was observed in all subgroups (Alex^®^—87%, Prolim^®^—83%, BiOSS^®^—73%). The highest rate of homogenous pattern was observed in the Alex while the lowest in the BiOSS LIM^®^ stent. Probably due to the small number of cases these differences were not statistically significant, however it suggests that StrCSA value stays in relation with neointimal proliferation pattern. In other words the most favorable profile was obtained in case of Alex^®^ stent, a little bit worse in Prolim^®^ stent i.e. in both fulfilling criterion of new generation DES. We found that also for thick strut BiOSS LIM^®^ stent this rate was lower comparing with Cypher stent (65%). This latter stent had a higher sirolimus concentration and considered as toxic mixture of polymers [25]. These abovementioned data are crucial since the homogeneous neointima pattern correlated in earlier reports with a high proportion of connective tissue and smooth muscle cells in histopathology indicating favorable vessel healing, whereas, heterogenous neointima was found to correlate with higher presence of fibrin as compared to homogenous one and was associated with poorer clinical outcomes [27].

## Study limitations

This registry has several limitations that should be acknowledged. First, the sample size was relatively small and no sample size calculation was performed. Other limitations of this study are its non-randomized manner and all known drawbacks of registry. Also, the significance of the new parameter ‘strut-cross sectional area’ should be verified in a larger group.

## Conclusions

In the QCA and OCT comparative analysis of three DES (Alex^®^, Prolim^®^ and BiOSS^®^) we found similar results for the first two, whereas a more pronounced response from the vessel wall was found in the BiOSS^®^ subgroup. Theoretically, the striking lack of differences between Alex^®^ and Prolim^®^ stents might be easily explained by a higher sirolimus concentration and more precise analysis of strut’s geometry represented by the proposed parameter—StrCSA that takes into account not only strut thickness but also its width.
